# The Value of Online Algorithms to Predict T-Cell Ligands Created by Genetic Variants

**DOI:** 10.1371/journal.pone.0162808

**Published:** 2016-09-12

**Authors:** Dyantha I. van der Lee, Margot J. Pont, J. H. Frederik Falkenburg, Marieke Griffioen

**Affiliations:** 1 Department of Hematology, Leiden University Medical Center, Leiden, The Netherlands; 2 Program in Immunology, Clinical Research Division, Fred Hutchinson Cancer Research Center, Seattle, Washington, United States of America; Hospital Israelita Albert Einstein, BRAZIL

## Abstract

Allogeneic stem cell transplantation can be a curative treatment for hematological malignancies. After HLA-matched allogeneic stem cell transplantation, beneficial anti-tumor immunity as well as detrimental side-effects can develop due to donor-derived T-cells recognizing polymorphic peptides that are presented by HLA on patient cells. Polymorphic peptides on patient cells that are recognized by specific T-cells are called minor histocompatibility antigens (MiHA), while the respective peptides in donor cells are allelic variants. MiHA can be identified by reverse strategies in which large sets of peptides are screened for T-cell recognition. In these strategies, selection of peptides by prediction algorithms may be relevant to increase the efficiency of MiHA discovery. We investigated the value of online prediction algorithms for MiHA discovery and determined the *in silico* characteristics of 68 autosomal HLA class I-restricted MiHA that have been identified as natural ligands by forward strategies in which T-cells from *in vivo* immune responses after allogeneic stem cell transplantation are used to identify the antigen. Our analysis showed that HLA class I binding was accurately predicted for 87% of MiHA of which a relatively large proportion of peptides had strong binding affinity (56%). Weak binding affinity was also predicted for a considerable number of antigens (31%) and the remaining 13% of MiHA were not predicted as HLA class I binding peptides. Besides prediction for HLA class I binding, none of the other online algorithms significantly contributed to MiHA characterization. Furthermore, we demonstrated that the majority of MiHA do not differ from their allelic variants in *in silico* characteristics, suggesting that allelic variants can potentially be processed and presented on the cell surface. In conclusion, our analyses revealed the *in silico* characteristics of 68 HLA class I-restricted MiHA and explored the value of online algorithms to predict T-cell ligands that are created by genetic variants.

## Introduction

Allogeneic stem cell transplantation (alloSCT) can be a curative treatment for hematological malignancies [1–2]. After HLA-matched alloSCT, a desired anti-tumor or graft-versus-leukemia (GvL) effect can be mediated by donor-derived T-cells recognizing polymorphic peptides in the context of HLA on the malignant cells of the patient. These polymorphic peptides or minor histocompatibility antigens (MiHA) arise as a result of differences in single nucleotide polymorphisms (SNP) in the genome between the recipient and stem cell donor [3–6]. These SNP differences often lead to a change in a single non-synonymous amino acid, resulting in presentation of the MiHA on the patient cell and expression of its allelic variant in the donor cell. Unfortunately, donor T-cells can also cause undesired graft-versus-host disease (GvHD) when MiHA are targeted that are expressed on healthy non-hematopoietic tissues [7–8]. Research focuses on characterization of MiHA with hematopoiesis-restricted expression, since donor T-cells for these MiHA attack the malignant cells of the patient, while sparing healthy hematopoietic cells of donor origin. As such, hematopoiesis-restricted MiHA can be used as targets for T-cell therapy to stimulate GvL reactivity without GvHD.

In 1995, HA-2 has been identified as first autosomal MiHA by mass spectrometry analysis of peptides eluted from HLA surface molecules [9]. Since then, methods for MiHA discovery developed in rapid succession and include screening of cDNA librariesand genetic approaches such as genetic linkage analysisand whole genome association scanning [4–6]. In these forward strategies, T-cells isolated from *in vivo* immune responses after alloSCT are used to identify MiHA and all peptides are thus characterized as natural T-cell ligands. Drawbacks of forward strategies are that large numbers of T-cells need to be isolated and expanded and that antigens have to be examined in detail for their tissue distribution to identify hematopoiesis-restricted MiHA with therapeutic relevance.

In reverse approaches, candidate MiHA encoded by genes with hematopoiesis-restricted expression can be selected to search for specific T-cells [10–12]. Selection of predefined antigens is frequently based on HLA class I binding affinity as predicted by online algorithms. A major drawback of reverse strategies is that many candidates cannot be confirmed as antigens that are endogenously processed and presented and recognized by specific T-cells. Inclusion of an additional step in which candidate antigens are selected for presence in the HLA-ligandome ensures endogenous processing and presentation, but does not guarantee that a donor T-cell exists with a T-cell receptor (TCR) that is capable of reacting with the antigen *in vivo*.

Similar to MiHA, neoantigens are peptides with amino acid changes that are recognized by specific T-cells [13–14]. In contrast to MiHA, neoantigens are created by tumor-specific mutations and can be targeted by autologous T-cells from the patient. In neoantigen discovery, tumor-specific mutations in coding exons as identified by whole exome or genome sequencing are searched for peptides with predicted binding to the HLA class I alleles as expressed by the patient and candidate neoantigens encoded by genes that are expressed in the tumor are selected to search for specific T-cells.

In reverse strategies for MiHA or neoantigen discovery, large sets of peptides need to be screened in order to discover antigens. Therefore, selection of peptides with predicted HLA class I binding affinity, peptide-HLA complex stability, proteasomal cleavage, affinity for the transporter associated with antigen processing and presentation (TAP) or *in vivo* immunogenicity may enhance the efficiency of antigen discovery. In this study, we explored the value of online prediction algorithms and determined the *in silico* characteristics for a set of 68 autosomal HLA class I-restricted MiHA that have been identified as natural T-cell ligands by forward approaches. We demonstrate that the algorithm for HLA class I binding accurately predicted 87% of MiHA of which a relatively large proportion (56%) are peptides with strong predicted binding to HLA class I. Besides prediction for HLA class I binding, none of the other online algorithms significantly contributed to MiHA characterization. We also demonstrate that the majority of MiHA do not differ from their allelic variants in *in silico* characteristics, suggesting that allelic variants can potentially be processed and presented on the cell surface and may therefore be relevant T-cell targets after alloSCT.

## Materials and Methods

### Minor histocompatibility antigens

A total of 68 autosomal HLA class I-restricted MiHA that have been identified as natural T-cell ligands by forward approaches have been included in the analyses. Epitopes which were restricted to multiple HLA-molecules (ACC-2D, LB-APOBEC3B-1K, LB-DHX33-1C, LB-GEMIN4-1V and UGT2B17) or length variants from a single epitope (LB-ERAP1-1R) were considered as different MiHA. Allelic variants exist for 60 MiHA and two MiHA (ACC-1Y and HB-1H) have allelic variants that have also been identified as *in vivo* T-cell targets [15–16]. For these two MiHA, the epitope that was first identified and published as *in vivo* T-cell target is indicated as MiHA, whereas its counterpart is indicated as allelic variant.

### Reference set of peptides

For accurate analysis of the value of online prediction algorithms for MiHA characterization, we composed a set of reference peptides. All peptides in the reference set were derived from 21 proteins for which HLA-A*02:01-restricted (n = 12) or HLA-B*07:02-restricted (n = 9) MiHA have been identified in the normal open reading frame. Whole protein sequences were screened for peptides with predicted binding to HLA-A*02:01 and HLA-B*07:02 using the online prediction algorithm NetMHCpan 2.8. Predicted strong and weak binding peptides as designated by default thresholds were included, leading to a total set of 1370 peptides of which 906 peptides were predicted to bind to HLA-A*02:01 and 464 peptides were predicted to bind to HLA-B*07:02.

### Online prediction algorithms

To predict binding affinity for HLA class I, stability of the peptide-HLA class I complex, proteasomal cleavage at the C-terminus, affinity for TAP and *in vivo* immunogenicity, the online available algorithms NetMHCpan 2.8[17], NetMHCstab 1.0 [18], NetChop 3.1 [19], TAPPred [20] and the MHC I Immunogenicity tool from the immune epitope database(IEDB) [21] were used, respectively. In addition, NetCTLpan 1.1 [22] was used to integrate predicted HLA class I binding affinity, C-terminal proteasomal cleavage and TAP transport efficiency.

In NetMHCpan 2.8, NetMHCstab 1.0 and NetChop 3.1, predictions are made using artificial neural networks (ANNs). ANNs of NetMHCpan 2.8 and NetMHCstab 1.0 have been trained for >150 and 10 different HLA-molecules based on >150.000 quantitative binding data and 5509 distinct peptide stability measurements, respectively. Predictions can be made for HLA-A or–B in NetMHCstab 1.0 and for HLA-A, -B, -C and -E in NetMHCpan 2.8. ANNs of the C-term 3.0 network of NetChop 3.1 have been trained on 1260 HLA class I ligands. In NetMHCpan 2.8, predictions are given as IC_50_ values in nM and %-Rank, which designates the rank of the predicted affinity of a certain epitope as compared to a set of 200.000 random natural peptides [17]. When using standard thresholds, epitopes are indicated as strong binding peptides (SB) if IC_50_≤50 nM or %-Rank≤0.5 and as weak binding peptides (WB) if IC_50_≤500 nM or %-Rank≤2. We defined peptides with IC_50_>500 nM and %-Rank>2 as non-binding peptides (NB). NetMHCstab 1.0 predicts the half-life of peptide-HLA class I complexes in hours [18]. The relative contribution of the predicted binding affinity of an epitope for its respective HLA-allele (as determined by NetMHCcons 1.0 [23]) to NetMHCstab 1.0 is 0.85 by default. Standard cut off values for highly stable complexes (HS) and weakly stable complexes (WS) are >6 hrs and >2 hrs, respectively. We indicated peptides with predicted stability ≤2 hrs as non-stable complexes (NS). Exact epitope sequences were fed into NetMHCpan 2.8 and NetMHCstab 1.0, whereas whole protein sequences were entered into NetChop 3.1. In NetChop 3.1, we used the C-term 3.0 network to predict C-terminal proteasomal cleavage [19]. Output is displayed as a score ranging 0–1, in which 0 signifies a low and 1 a high likelihood of proteasomal cleavage. The standard threshold for proteasomal cleavage is a score >0.5.

Affinity for the TAP transporter was predicted by TAPPred [20]. TAPPred uses a support vector machine (SVM)-based method which is trained on experimentally determined IC_50_ values of 431 peptides that bind to TAP with different affinities. The cascade SVM-based method was used to predict TAP transporter affinity. Output is given as a scale on which a score of 0 corresponds to a normalized IC_50_>1000 nM and a score of 10 to a normalized IC_50_<0.003 nM. Exact peptide sequences were entered into the algorithm and, using standard cut off values, were divided in high (>6), intermediate (>3) or low (≤3) affinity peptides for the TAP transporter.

NetCTLpan 1.1 predicts T-cell epitopes in protein sequences based on an integrated approach of HLA class I binding affinity, C-terminal proteasomal cleavage and TAP transport efficiency as predicted by NetMHC pan 2.3, the C-term 3.0 network of NetChop 3.0 and a weight matrix based method, respectively [22]. In this algorithm, the default weight on HLA class I binding affinity, C-terminal proteasomal cleavage and TAP transport efficiency is 0.750, 0.225 and 0.025, respectively. Predictions are given as %-Rank, which designates the rank of the predicted affinity of a certain epitope as compared to a set of 200.000 random natural peptides. Whole protein sequences were entered into the algorithm and the standard threshold of %-Rank<1 was used for epitope identification.

Epitope immunogenicity, which is defined as the ability of a certain epitope to be recognized by a specific TCR, was determined by the MHC I Immunogenicity tool from the Immune Epitope Database and Analysis Resource (IEDB) [21]. Exact peptide sequences were entered and thresholds for *in vivo* immunogenicity with 90% specificity were determined for HLA-A*02:01 (>0.27) and HLA-B*07:02 (>0.22) based on analysis of prediction data for MiHA and reference peptides by receiver operating characteristics (ROC) curves. In the IEDB tool, amino acid residues at anchor positions are masked to avoid bias by HLA class I binding affinity according to binding motifs as available in http://www.cbs.dtu.dk/biotools/MHCMotifViewer/Human_alleles.html [24].

### Statistical analysis

Fisher’s exact test was used to compare *in silico* characteristics between MiHA and reference peptides and to compare predicted C-terminal proteasomal cleavage and *in vivo* immunogenicity between MiHA and their allelic variants. For comparison of predicted HLA class I binding affinity, stability of the peptide-HLA class I complexes and *in vivo* immunogenicity between MiHA and their allelic variants, Wilcoxon signed rank test was used. P-values <0.05 were considered significant. ROC curves were plotted to determine the sensitivity and specificity of default thresholds and to define the thresholds for the MHC I Immunogenicity tool from the IEDB. The performance of the online algorithms was evaluated by the area under the ROC curve (AUC) in which p-values <0.05 were considered significant.

## Results

### HLA class I-restricted minor histocompatibility antigens

HLA class I-restricted MiHA that have been identified by forward approaches are antigens that are targeted by T-cells *in vivo*. These natural T-cell ligands follow by definition all rules that are required for endogenous processing and presentation and antigen recognition by specific T-cells. We therefore selected these antigens to explore the value of online available tools for prediction of HLA class I binding affinity, stability of the peptide-HLA class I complex, proteasomal cleavage, TAP transporter affinity and *in vivo* immunogenicity. Autosomal HLA class I-restricted MiHA that have been identified by forward approaches (reviewed by Griffioen et al.[6], [25] and unpublished work) are listed in Table 1 (n = 68) and the results of *in silico* analyses are shown in Table 2. Of the 68 MiHA as shown in Table 1, 34 antigens are 9-mer peptides, 17 antigens are 10-mer peptides and 16 antigens are 11-mer peptides. LB-NADK-1K is the only peptide of 13 amino acids in length. Overall, the 68 MiHA bind to 19 different HLA class I-alleles, of which HLA-A*02:01 (n = 17) and HLA-B*07:02 (n = 18) are most frequent.

**Table 1:** HLA class I-restricted minor histocompatibility antigens.

MiHA name	Sequence	Length	Gene	SNP	HLA-allele
ACC-1Y	DYLQ[**Y**/C]VLQI	9	*BCL2A1*	rs1138357	A*24:02
ACC-2D	KEFED[**D**/G]IINW	10	*BCL2A1*	rs3826007	B*44:02
ACC-2D	KEFED[**D**/G]IINW	10	*BCL2A1*	rs3826007	B*44:03
ACC-6	MEIFIEVFSHF	11	*HMSD*	rs9945924	B*44:03
CTSH(R)/A31	ATLPLLCA[**R**/G]	9	*CTSH*	rs2289702	A*31:01
CTSH(R)/A33	WATLPLLCA[**R**/G]	10	*CTSH*	rs2289702	A*33:03
DPH1	S[**V**/L]LPEVDVW	9	*DPH1*	rs35394823	B*57:01
HA-1	VL[**H**/R]DDLLEA	9	*HMHA1*	rs1801284	A*02:01
HA-2	YIGEVLVS[**V**/M]	9	*MYO1G*	rs61739531	A*02:01
HA-3T	V[**T**/M]EPGTAQY	9	*AKAP13*	rs2061821	A*01:01
HA-8R	[**R**/P]TLDKVLEV	9	*KIAA0020*	rs2173904	A*02:01
HB-1H	EEKRGSL[**H**/Y]VW	10	*HMHB1*	rs161557	B*44:03
HEATR1-1E	ISKERA[**E**/G]AL	9	*HEATR1*	rs2275687	B*08:01
HwA11-S	CIPPD[**S**/T]LLFPA	11	*C19ORF48*	rs3745526	A*02:01
LB-ADIR-1F	SVAPALAL[**F**/S]PA	11	*TOR3A*	rs2296377	A*02:01
LB-APOBEC3B-1K	[**K**/E]PQYHAEMCF	10	*APOBEC3B*	rs2076109	B*07:02
LB-APOBEC3B-1K	[**K**/E]PQYHAEMCF	10	*APOBEC3B*	rs2076109	B*08:01
LB-ARHGDIB-1R	LPRACW[**R**/P]EA	9	*ARHGDIB*	rs4703	B*07:02
LB-BCAT2-1R	QP[**R**/T]RALLFVIL	11	*BCAT2*	rs11548193	B*07:02
LB-C16ORF-1R	[**R**/W]PCPSVGLSFL	11	*C16ORF*	rs305087	B*07:02
LB-C19ORF48-2E	TAWPGAP[**E**/G]V	9	*C19ORF48*	rs4801853	B*51:01
LB-CCL4-1T	CADPSE[**T**/S]WV	9	*CCL4*	rs1719152	A*02:01
LB-CLYBL-1Y	SLAA[**Y**/D]IPRL	9	*CLYBL*	rs17577293	A*02:01
LB-CYBA-1Y	STMERWGQK[**Y**/H]	10	*CYBA*	rs4673	A*01:01
LB-DHX33-1C	YLYEGGIS[**C**/R]	9	*DHX33*	rs8069315	A*02:01
LB-DHX33-1C	YLYEGGIS[**C**/R]	9	*DHX33*	rs8069315	C*03:03
LB-EBI3-1I	RPRARYY[**I**/V]QV	10	*EBI3*	rs4740	B*07:02
LB-ECGF-1H	RP[**H**/R]AIRRPLAL	11	*TYMP*	rs112723255	B*07:02
LB-ERAP1-1R	HP[**R**/P]QEQIAL	9	*ERAP1*	rs26653	B*07:02
LB-ERAP1-1R	HP[**R**/P]QEQIALLA	11	*ERAP1*	rs26653	B*07:02
LB-FUCA2-1V	RLRQ[**V**/M]GSWL	9	*FUCA2*	rs3762002	B*07:02
LB-GEMIN4-1V	FPALRFVE[**V**/E]	9	*GEMIN4*	rs4968104	B*07:02
LB-GEMIN4-1V	FPALRFVE[**V**/E]	9	*GEMIN4*	rs4968104	B*08:01
LB-GLE1-1V	GQ[**V**/I]RLRALY	9	*GLE1*	rs2275260	B*15:01
LB-GSTP1-1V	DLRCKY[**V**/I]SL	9	*GSTP1*	rs1695	B*08:01
LB-ITGB2-1	GQAGFFPSPF	10	*ITGB2*	rs760462	B*15:01
LB-MOB3A-1C	[**C**/S]PRPGTWTC	9	*MOB3A*	rs11541046	B*07:02
LB-NADK-1K	AVHNGLGE[**K**/N]GSQA	13	*NADK*	rs4751	A*03:01
LB-NCAPD3-1Q	WL[**Q**/R]GVVPVV	9	*NCAPD3*	rs12292394	A*02:01
LB-NDC80-1P	HLEEQI[**P**/A]KV	9	*NDC80*	rs9051	A*02:01
LB-NUP133-1R	SEDLILC[**R**/Q]L	9	*NUP133*	rs1065674	B*40:01
LB-OAS1-1R	ETDDPR[**R**/T]YQKY	11	*OAS1*	rs1051042	A*01:01
LB-PDCD11-1F	GPDSSKT[**F**/L]LCL	11	*PDCD11*	rs2986014	B*07:02
LB-PFAS-1P	A[**P**/S]GHTRRKL	9	*PFAS*	rs9891699	B*07:02
LB-PNP-1S	TQAQIFDY[**S**/G]EI	11	*PNP*	rs1049564	B*13:02
LB-PRCP-1D	FMWDVAE[**D**/E]LKA	11	*PRCP*	rs2298668	A*02:01
LB-SON-1R	SETKQ[**R**/C]TVL	9	*SON*	rs13047599	B*40:01
LB-SRGN-1R	ESSVQGYPT[**R**/Q]R	11	*SRGN*	rs2805910	A*68:01
LB-SSR1-1S	[**S**/L]LAVAQDLT	9	*SSR1*	rs10004	A*02:01
LB-SWAP70-1Q	MEQLE[**Q**/E]LEL	9	*SWAP70*	rs415895	B*40:01
LB-TMEM8A-1I	RPRSVT[**I**/V]QPLL	11	*TMEM8A*	rs2071915	B*07:02
LB-TRIP10-1EPC	G[**E**/G][**P**/S]QDL[**C**/G]TL	9	*TRIP10*	rs1049229, rs1049230, rs1049232	B*40:01
LB-TTK-1D	RLH[**D**/E]GRVFV	9	*TTK*	rs240226	A*02:01
LB-USP15-1I	MPSHLRN[**I**/T]LL	10	*USP15*	rs11174420	B*07:02
LB-WNK1-1I	RTLSPE[**I**/M]ITV	10	*WNK1*	rs12828016	A*02:01
LB-ZDHHC6-1Y	RPR[**Y**/H]WILLVKI	11	*ZDHHC6*	rs4918752	B*07:02
LB-ZNFX1-1Q	NEIEDVW[**Q**/H]LDL	11	*ZNFX1*	rs238221	B*40:01
LRH-1	TPNQRQNVC	9	*P2RX5*	rs3215407	B*07:02
P2RX7	WFHHC[**H**/R]PKY	9	*P2RX7*	rs7958311	A*29:02
PANE1	RVWDLPGVLK	10	*CENPM*	rs5758511	A*03:01
SLC1A5	AE[**A**/P]TANGGLAL	11	*SLC1A5*	rs3027956	B*40:02
SP110	SLP[**R**/G]GTSTPK	10	*SP110*	rs1365776	A*03:01
TRIM22	MAVPPC[**C**/R]IGV	10	*TRIM22*	rs187416296	A*02:01
UGT2B17	CVATMIFMI	9	*UGT2B17*	Gene deletion	A*02:01
UGT2B17	AELLNIPFLY	10	*UGT2B17*	Gene deletion	A*29:02
UGT2B17	AELLNIPFLY	10	*UGT2B17*	Gene deletion	B*44:03
UTA2-1	QL[**L**/P]NSVLTL	9	*KIAA1551*	rs2166807	A*02:01
ZAPHIR	IPRDSWWVEL	10	*ZNF419*	rs2074071	B*07:02

Polymorphic amino acids are shown in brackets. Amino acids as present in MiHA are indicated in bold.

MiHA for which the allelic variant has also been identified as *in vivo* T-cell target. The epitope which was first identified and published as *in vivo* T-cell target is indicated as MiHA, whereas its counterpart is indicated as allelic variant.

SNP does not directly encode MiHA but creates a new protein from which MiHA is derived.

SNP encoding MiHA in an alternative reading frame.

**Table 2:** *In silico* analysis of minor histocompatibility antigens.

MiHA name	Sequence	HLA-allele	NetMHCpan 2.8 (nM/%-Rank)	NetMHC stab 1.0 t½ (hrs)	NetChop 3.1	TAPPred	NetCTL pan 1.1 (%-Rank)	IEDB
ACC-1Y	DYLQ[**Y**/C]VLQI	A*24:02	82.21/0.40	5.87	0.91	5.01	0.20	-0.16
ACC-2D	KEFED[**D**/G]IINW	B*44:02	72.66/0.12	n.a.	0.41	8.41	0.30	0.42
ACC-2D	KEFED[**D**/G]IINW	B*44:03	35.30/0.10	n.a.	0.41	8.41	0.15	0.42
ACC-6	MEIFIEVFSHF	B*44:03	64.04/0.15	n.a.	0.96	7.71	0.01	0.42
CTSH(R)/A31	ATLPLLCA[**R**/G]	A*31:01	17.34/0.30	n.a.	0.27	3.84	0.30	-0.06
CTSH(R)/A33	WATLPLLCA[**R**/G]	A*33:03	194.66/1.50	n.a.	0.27	4.00	2.00	-0.05
DPH1	S[**V**/L]LPEVDVW	B*57:01	77.67/0.25	n.a.	0.45	5.80	0.80	0.16
HA-1	VL[**H**/R]DDLLEA	A*02:01	21.96/1.00	1.70	0.96	6.96	0.80	0.09
HA-2	YIGEVLVS[**V**/M]	A*02:01	5.92/0.20	7.96	0.97	7.95	0.10	0.08
HA-3T	V[**T**/M]EPGTAQY	A*01:01	30.55/0.08	4.11	0.98	5.80	0.05	0.02
HA-8R	**[R**/P]TLDKVLEV	A*02:01	80.99/2.00	2.49	0.97	4.17	1.00	-0.10
HB-1H	EEKRGSL[**H**/Y]VW	B*44:03	98.36/0.25	n.a.	0.90	5.84	0.80	-0.11
HEATR1-1E	ISKERA[**E**/G]AL	B*08:01	588.87/1.50	n.a.	0.70	6.12	1.50	0.24
HwA11-S	CIPPD[**S**/T]LLFPA	A*02:01	829.68/6.00	0.50	0.81	7.19	5.00	-0.08
LB-ADIR-1F	SVAPALAL[**F**/S]PA	A*02:01	50.89/1.50	0.79	0.91	8.35	1.00	0.15
LB-APOBEC3B-1K	[**K**/E]PQYHAEMCF	B*07:02	215.46/1.00	2.11	0.26	6.97	1.50	-0.06
LB-APOBEC3B-1K	[**K**/E]PQYHAEMCF	B*08:01	6146.92/9.00	n.a.	0.26	6.97	16.00	-0.05
LB-ARHGDIB-1R	LPRACW[**R**/P]EA	B*07:02	40.16/0.40	2.12	0.42	-0.73	0.80	0.31
LB-BCAT2-1R	QP[**R**/T]RALLFVIL	B*07:02	20.34/0.17	4.14	0.94	7.26	0.20	0.31
LB-C16ORF-1R	[**R**/W]PCPSVGLSFL	B*07:02	93.99/0.80	2.83	0.90	5.62	0.40	-0.20
LB-C19ORF48-2E	TAWPGAP[**E/**G]V	B*51:01	n.a./0.50	n.a.	0.97	4.05	0.80	0.18
LB-CCL4-1T	CADPSE[**T**/S]WV	A*02:01	5048.30/15.00	0.35	0.29	4.89	32.00	0.09
LB-CLYBL-1Y	SLAA[**Y**/D]IPRL	A*02:01	4.28/0.12	6.41	0.97	3.94	0.05	0.19
LB-CYBA-1Y	STMERWGQK[**Y**/H]	A*01:01	243.43/0.25	0.61	0.79	4.08	0.15	0.17
LB-DHX33-1C	YLYEGGIS[**C**/R]	A*02:01	24.03/1.00	3.31	0.04	8.31	1.50	0.18
LB-DHX33-1C	YLYEGGIS[**C**/R]	C*03:03	1662.22/7.00	n.a.	0.04	8.31	50.00	0.18
LB-EBI3-1I	RPRARYY[**I**/V]QV	B*07:02	11.77/0.10	5.89	0.96	8.40	0.15	0.14
LB-ECGF-1H	RP[**H**/R]AIRRPLAL	B*07:02	2.49/0.01	6.71	0.92	5.86	0.01	0.28
LB-ERAP1-1R/9-mer	HP[**R**/P]QEQIAL	B*07:02	4.63/0.03	5.65	0.96	0.76	0.05	0.02
LB-ERAP1-1R/11-mer	HP[**R**/P]QEQIALLA	B*07:02	107.63/0.80	1.97	0.12	4.45	1.50	0.04
LB-FUCA2-1V	RLRQ[**V**/M]GSWL	B*07:02	78.61/0.80	1.48	0.90	4.07	0.40	-0.04
LB-GEMIN4-1V	FPALRFVE[**V**/E]	B*07:02	32.34/0.30	2.99	0.98	1.42	0.30	0.19
LB-GEMIN4-1V	FPALRFVE[**V**/E]	B*08:01	21.52/0.08	n.a.	0.98	1.42	0.05	0.26
LB-GLE1-1V	GQ[**V**/I]RLRALY	B*15:01	61.54/0.80	7.74	0.88	3.84	1.00	0.13
LB-GSTP1-1V	DLRCKY[**V**/I]SL	B*08:01	9.92/0.03	n.a.	0.77	5.41	0.05	-0.12
LB-ITGB2-1	GQAGFFPSPF	B*15:01	10.28/0.05	9.37	0.54	8.62	0.80	0.12
LB-MOB3A-1C	[**C**/S]PRPGTWTC	B*07:02	470.83/1.50	3.59	0.70	8.46	1.50	0.28
LB-NCAPD3-1Q	WL[**Q**/R]GVVPVV	A*02:01	10.56/0.50	6.59	0.91	-1.08	0.20	0.09
LB-NDC80-1P	HLEEQI[**P**/A]KV	A*02:01	70.32/2.00	3.15	0.97	7.62	1.50	0.01
LB-NUP133-1R	SEDLILC[**R**/Q]L	B*40:01	139.60/0.80	1.22	0.91	3.84	0.40	0.10
LB-OAS1-1R	ETDDPR[**R**/T]YQKY	A*01:01	133.89/0.15	2.85	0.85	6.84	0.10	-0.11
LB-PDCD11-1F	GPDSSKT[**F**/L]LCL	B*07:02	585.68/1.50	1.38	0.97	5.70	1.50	-0.42
LB-PFAS-1P	A[**P**/S]GHTRRKL	B*07:02	19.95/0.17	3.03	0.94	6.12	0.30	0.05
LB-PNP-1S	TQAQIFDY[**S**/G]EI	B*13:02	n.a./0.80	n.a.	0.57	6.47	0.80	0.08
LB-PRCP-1D	FMWDVAE[**D**/E]LKA	A*02:01	11.09/0.50	2.51	0.06	7.80	1.50	0.16
LB-SON-1R	SETKQ[**R**/C]TVL	B*40:01	98.73/0.80	1.37	0.92	3.85	0.80	-0.21
LB-SRGN-1R	ESSVQGYPT[**R**/Q]R	A*68:01	60.54/1.50	n.a.	0.79	4.10	0.40	-0.04
LB-SWAP70-1Q	MEQLE[**Q**/E]LEL	B*40:01	24.68/0.20	1.06	0.95	7.40	0.15	-0.01
LB-TMEM8A-1I	RPRSVT[**I**/V]QPLL	B*07:02	6.88/0.05	6.30	0.97	7.18	0.05	-0.07
LB-TRIP10-1EPC	G[**E**/G][**P**/S]QDL[**C**/G]TL	B*40:01	271.70/1.00	1.51	0.96	7.14	0.80	-0.13
LB-TTK-1D	RLH[**D**/E]GRVFV	A*02:01	30.45/1.50	5.32	0.89	4.01	0.80	0.22
LB-USP15-1I	MPSHLRN[**I**/T]LL	B*07:02	11.79/0.10	2.42	0.66	5.05	0.20	0.12
LB-WNK1-1I	RTLSPE[**I**/M]ITV	A*02:01	388.11/4.00	1.34	0.97	6.76	1.50	0.18
LB-ZDHHC6-1Y	RPR[**Y**/H]WILLVKI	B*07:02	31.86/0.30	3.17	0.97	4.03	0.30	0.25
LB-ZNFX1-1Q	NEIEDVW[**Q**/H]LDL	B*40:01	51.33/0.40	0.85	0.94	4.37	0.30	0.32
LRH-1	TPNQRQNVC	B*07:02	1219.83/3.00	1.61	0.13	-0.88	16.00	-0.16
P2RX7	WFHHC[**H**/R]PKY	A*29:02	29.98/0.50	n.a.	0.96	4.61	0.40	-0.11
PANE1	RVWDLPGVLK	A*03:01	45.68/0.25	2.91	0.56	6.78	0.40	0.13
SLC1A5	AE[**A**/P]TANGGLAL	B*40:02	40.81/0.40	n.a.	0.96	4.02	0.10	0.16
SP110	SLP[**R**/G]GTSTPK	A*03:01	405.03/1.50	1.47	0.98	6.09	0.80	-0.01
UGT2B17	CVATMIFMI	A*02:01	432.65/4.00	0.96	0.10	3.89	5.00	0.00
UGT2B17	AELLNIPFLY	A*29:02	509.05/3.00	n.a.	0.97	3.90	1.50	0.19
UGT2B17	AELLNIPFLY	B*44:03	22.25/0.05	n.a.	0.97	3.90	0.05	0.19
UTA2-1	QL[**L**/P]NSVLTL	A*02:01	92.30/2.00	4.61	0.98	3.99	1.50	-0.12
ZAPHIR	IPRDSWWVEL	B*07:02	10.21/0.10	4.42	0.97	3.84	0.05	0.40

Polymorphic amino acids are shown in brackets. Amino acids as present in MiHA are indicated in bold.

n.a., HLA class I allele not available in the algorithm.

### HLA class I binding affinity

**Fig 1:**
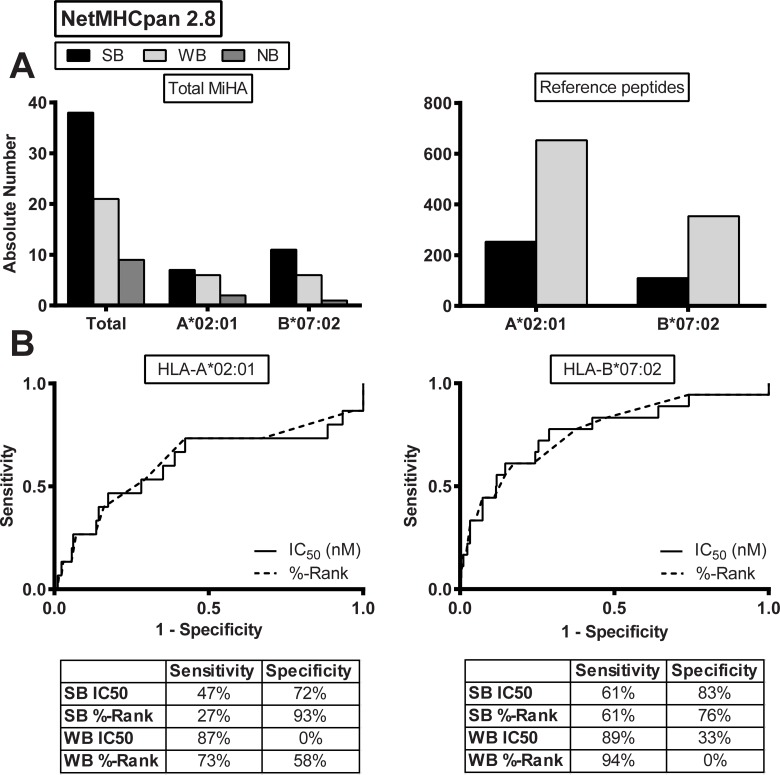
Predicted HLA class I binding affinity. (A) HLA class I binding affinity as predicted by NetMHCpan 2.8 for MiHA that have been identified as natural T-cell ligands by forward strategies. Results are shown for the total group of MiHA (n = 68) and for HLA-A*02:01-restricted MiHA (n = 15) and HLA-B*07:02-restricted MiHA (n = 18) (left) as compared to reference peptides with predicted binding affinity to HLA-A*02:01 (n = 906) or HLA-B*07:02 (n = 464) (right). Indicated are absolute numbers of peptides with strong predicted binding (SB; black bars), weak predicted binding (WB; light grey bars) and non-binding (NB; dark grey bars).The data show that the proportion of SB peptides in the group of MiHA is higher than in the reference set of peptides (54% *versus* 28% with p = 0.0581 for HLA-A*02:01; 65% *versus* 24% with p = 0.0005 for HLA-B*07:02 using Fisher’s exact test). (B) ROC curves for HLA class I binding affinity as predicted by NetMHCpan 2.8 for HLA-A*02:01 (left) and HLA-B*07:02 (right). Sensitivity and 1-specificity are shown on the Y- and X-axis, respectively. Curves for IC_50_ (solid line) and %-Rank (dashed line) are plotted based on prediction data for MiHA and reference peptides. Sensitivity and specificity are indicated for default values for SB (≤0.5%-Rank or IC_50_≤50 nM) and WB (≤2%-Rank or IC_50_≤500 nM). For HLA-A*02:01, AUC values for %-Rank and IC_50_ are 0.625 and 0.609, respectively (p = 0.0964 for %-Rank; p = 0.1486 for IC_50_). For HLA-B*07:02, AUC values for %-Rank and IC_50_ are 0.767 and 0.765, respectively (p = 0.0001 for %-Rank; p = 0.0001 for IC_50_).

Binding of antigens to HLA class I is a strict requirement for provoking CD8 T-cell responses. Therefore, we predicted HLA class I binding affinity by NetMHCpan 2.8 and compared the characteristics of MiHA with a reference set of peptides (Fig 1A). When using standard thresholds to designate strong binding peptides (SB; ≤0.5%-Rank or IC_50_≤50 nM) and weak binding peptides (WB; ≤2%-Rank or IC_50_≤500 nM), 38 (56%) antigens of the total set of 68 MiHA were predicted as SB peptides, 21 (31%) antigens as WB peptides and 9 (13%) antigens as NB peptides. For 3 MiHA that have been identified as NB peptides (LB-NADK-1K, LB-SSR1-1S and TRIM22), it is unclear whether the peptide as reported in Table 1 is the actual minimal epitope. For LB-NADK-1K and TRIM22, other 9-11-mer peptide variants can be found with weak predicted binding to HLA-A*03:01 and HLA-A*02:01, respectively, but T-cell recognition of these peptides has not been tested (LB-NADK-1K; own observations) or reported (TRIM22; [26]). For LB-SSR1-1S, no other 9-11-mer peptide variant with predicted HLA binding can be found, but T-cell recognition of the peptide as reported in Table 1 requires concentrations >5.000 nM, suggesting that the peptide may not be the actual minimal epitope [27]. These 3 MiHA were excluded from our dataset, resulting in a total number of 65 HLA class I-restricted MiHA containing 15 antigens that bind to HLA-A*02:01 and 18 antigens that bind to HLA-B*07:02 that were used for further analyses to determine the value of online prediction algorithms. Analysis of the 6 remaining NB peptides revealed that 5 (83%) antigens contain a cysteine residue either as anchor (n = 2) or as residue adjacent to the anchor (n = 3). In contrast, only 13 (22%) of the 59 MiHA that are predicted as SB or WB peptides contain a cysteine residue and of these 13 antigens, only 6 peptides contained the cysteine residue as anchor (n = 1) or as residue adjacent to the anchor (n = 5). The data suggest that NetMHCpan 2.8 is less accurate in predicting binding affinity for peptides with cysteine residues at anchor or adjacent positions.

For accurate analysis of the value of online prediction algorithms for MiHA characterization, we compared prediction data for HLA-A*02:01- and HLA-B*07:02-restricted MiHA with a reference set of peptides. Of the 15 HLA-A*02:01-restricted MiHA, 13 antigens were predicted to bind to HLA-A*02:01 of which 7 (54%) antigens were SB peptides and 6 (46%) antigens were WB peptides. The remaining 2 antigens were NB peptides. Of the 18 HLA-B*07:02-restricted MiHA, 17 antigens were predicted to bind to HLA-B*07:02 of which 11 (65%) antigens were SB peptides and 6 (35%) antigens were WB peptides. Only one HLA-B*07:02-restricted MiHA was a NB peptide. The reference set consisted of 1370 peptides (9-11-mer peptides) with predicted binding to HLA-A*02:01 (n = 906) or HLA-B*07:02 (n = 464) as determined by NetMHCpan 2.8 using default thresholds. All peptides were derived from 21 proteins for which HLA-A*02:01-restricted MiHA (n = 12) or HLA-B*07:02-restricted MiHA (n = 9) have been identified in the normal open reading frame. Analysis revealed that 26% of the reference peptides were SB peptides and 74% were WB peptides. The percentages SB and WB peptides in the reference set were comparable for HLA-A*02:01 and HLA-B*07:02 (28% SB and 72% WB peptides for HLA-A*02:01; 24% SB and 76% WB peptides for HLA-B*07:02). As expected, the proportion of SB peptides in the group of MiHA is higher than in the reference set of peptides and this difference is more pronounced for HLA-B*07:02 (65% *versus* 24%; p = 0.0005) than for HLA-A*02:01 (54% *versus* 28%; p = 0.0581). In addition, NetMHCpan 2.8 failed to predict HLA class I binding for 2 (13%) HLA-A*02:01-restricted MiHA (HwA11-S CIPPDSLLFPA; LB-CCL4-1T CADPSETWV) and one (6%) HLA-B*07:02-restricted MiHA (LRH-1 TPNQRQNVC).

To evaluate the performance of NetMHCpan 2.8, ROC curves were plotted separately for HLA-A*02:01 and HLA-B*07:02 (Fig 1B). For both HLA restriction alleles, comparison of the overall performance of %-Rank and IC_50_ revealed similar AUC values. Furthermore, sensitivity and specificity values for default thresholds for SB and WB peptides demonstrated that MiHA are characterized with low sensitivity but high specificity by selecting SB peptides, whereas selection of WB peptides leads to characterization of MiHA with high sensitivity but low specificity.

### Stability of the peptide-HLA class I complex

**Fig 2:**
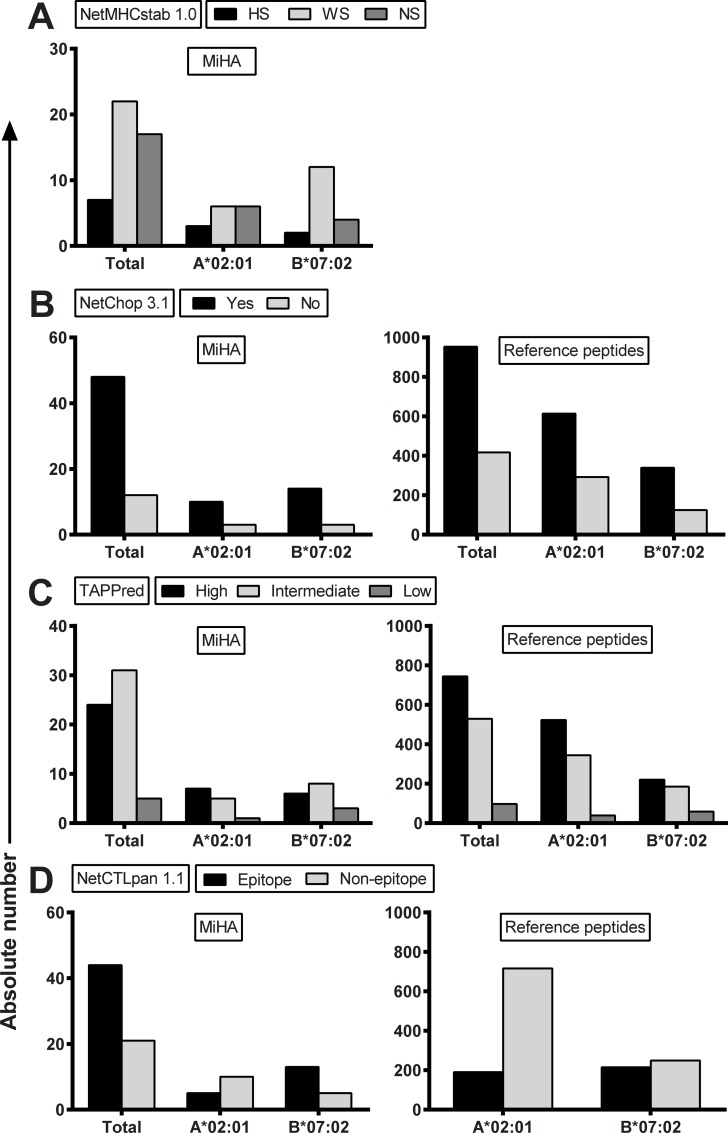
Predicted stability of the peptide-HLA class I complex, proteasomal cleavage, TAP transport and their integration. (A) Peptide-HLA class I complex stability as predicted by NetMHCstab 1.0 with default settings for all MiHA for which HLA class I restriction alleles are available in the algorithm (n = 46), HLA-A*02:01-restricted MiHA (n = 15) and HLA-B*07:02-restricted MiHA (n = 18). Indicated are absolute numbers of MiHA that are predicted as highly stable (HS; black bars), weakly stable (WS; light grey bars) or non-stable (NS; dark grey bars) complexes. The data show that NetMHCstab 1.0 accurately predicted 29 of the 46 MiHA, including 9 HLA-A*02:01-restricted MiHA and 14 HLA-B*07:02-restricted MiHA. (B) Proteasomal cleavage at the C-terminus as predicted by NetChop 3.1 for all different MiHA peptides (n = 60) and for MiHA that are predicted to bind to HLA-A*02:01 (n = 13) or HLA-B*07:02 (n = 17) by NetMHCpan 2.8. Whole protein sequences were fed into the algorithm and default settings were used to predict proteasomal cleavage. Indicated are absolute numbers of peptides with predicted cleavage at the C-terminus for MiHA (left) and the reference set of peptides (right). No significant difference was observed in proportion of peptides with predicted cleavage at the C-terminus between MiHA and reference peptides (80% for MiHA *versus* 70% for reference peptides, p = 0.3141 using Fisher’s exact test). (C) Affinity for the TAP transporter as predicted by TAPPred with default settings for all different MiHA peptides (n = 60) and for MiHA that are predicted to bind to HLA-A*02:01 (n = 13) or HLA-B*07:02 (n = 17) by NetMHCpan 2.8. Indicated are absolute numbers of peptides with high (black bars), intermediate (light grey bars) and low (dark grey bars) affinity for TAP for the MiHA (left) and the reference peptides (right). No significant difference was observed in proportion of peptides with high or weak affinity for TAP between MiHA (43% high, 43% intermediate and 13% low affinity) and the reference peptides (54% high, 39% intermediate and 7% low affinity). (D) Epitope prediction by NetCTLpan 1.1 with default settings for the total set of MiHA (n = 65) and for HLA-A*02:01-restricted MiHA (n = 15) and HLA-B*07:02-restricted MiHA (n = 18) (left) as compared to reference peptides (right). Indicated are absolute numbers of peptides that are predicted as epitopes (black bars) or non-epitopes (grey bars). For HLA-A*02:01, the proportion of peptides that are predicted as T-cell epitopes is similar between MiHA and reference peptides (33% *versus* 21%, p = 0.3338), whereas for HLA-B*07:02, the proportion of peptides that are predicted as T-cell epitopes is higher for MiHA than for reference peptides although it did not reach statistical significance (72% *versus* 46%, p = 0.0514).

Since NetMHCpan 2.8 failed to predict 6 MiHA (including 2 HLA-A*02:01-restricted MiHA and one HLA-B*07:02-restricted MiHA) using default settings, we explored whether MiHA are more accurately predicted as stable peptide-HLA class I complexes by NetMHCstab 1.0 (Fig 2A). In NetMHCstab 1.0, HLA class I restriction alleles were available for 46 MiHA as shown in Table 2 and we therefore restricted the analysis to HLA-A*02:01- and HLA-B*07:02-restricted MiHA. Of the 15 HLA-A*02:01-restricted MiHA, 3 (20%) antigens were predicted as highly stable complexes (HS; half-life>6 hrs), 6 (40%) antigens as weakly stable complexes (WS; >2 hrs and ≤6 hrs) and 6 (40%) antigens as non-stable complexes (NS;≤2 hrs). Of the 18 HLA-B*07:02-restricted MiHA, 2 (11%), 12 (67%) and 4 (22%) antigens were predicted as HS, WS and NS complexes, respectively. In conclusion, MiHA are predicted as HLA class I binding peptides by NetMHCpan 2.8 with higher sensitivity than as stable peptide-HLA complexes by NetMHCstab 1.0.

In NetMHCstab 1.0, stability predictions are based for 85% on HLA class I binding affinity as predicted by the online algorithm NetMHCcons 1.0. ROC curves were plotted for NetMHCstab 1.0 and NetMHCcons 1.0 separately for HLA-A*02:01 and HLA-B*07:02 (S1 Fig). Comparison of AUC showed that NetMHCstab 1.0 is slightly superior to NetMHCcons 1.0 for HLA-B*07:02, but not for HLA-A*02:01. Moreover, ROC analysis confirmed the low sensitivity of NetMHCstab 1.0 to characterize MiHA, but demonstrated that the specificity of the algorithm is high.

### Proteasomal cleavage

Surface presentation of an antigen by HLA class I requires intracellular processing of the protein by the proteasome. It has been demonstrated that the exact C-terminus of an antigenic peptide is generated by the proteasome, whereas the N-terminus is trimmed by amino peptidases [28]. We investigated the presence of a proteasomal cleavage site at the exact C-terminus of the MiHA by NetChop 3.1 (Fig 2B). Whole protein sequences were fed into the algorithm and MiHA that are presented by two different HLA class I restriction alleles (ACC-2D, LB-APOBEC3B-1K, LB-DHX33-1C, LB-GEMIN4-1V and UGT2B17) were analyzed only once, resulting in a total number of 60 different MiHA that were examined. Of the 60 MiHA, 48 (80%) antigens were predicted to be cleaved immediately after the C-terminal amino acid. Since proteasomal cleavage is not influenced by HLA class I binding affinity, we compared predicted proteasomal cleavage between MiHA and reference peptides using the same sets as described above. Of the 30 MiHA with predicted binding to HLA-A*02:01 or HLA-B*07:02, 24 (80%) antigens were predicted to be cleaved after the C-terminus as compared to 70% of the reference peptides, indicating that predicted proteasomal cleavage by NetChop 3.1 is similar between MiHA and reference peptides (sensitivity 80% and specificity 30% by ROC analysis).

### TAP transporter affinity

Transport of peptides from the cytosol into the endoplasmic reticulum occurs via the TAP transporter and can be predicted by TAPPred (Fig 2C). Of the 60 different MiHA, 24 (40%) antigens were predicted as peptides with high binding affinity for TAP (>6), 31 (52%) antigens as peptides with intermediate binding affinity (>3) and 5 (8%) antigens as peptides with low binding affinity (≤3). Similar as for proteasomal cleavage, TAP affinity is not influenced by HLA class I binding affinity and we therefore compared the same set of MiHA and reference peptides. Analysis of the 30 MiHA with predicted binding to HLA-A*02:01 or HLA-B*07:02 revealed that 13 (43%) epitopes had high binding affinity, 13 (43%) epitopes had intermediate binding affinity and 4 (13%) epitopes had low binding affinity for TAP. Similar percentages were predicted for peptides in the reference set (54% high, 39% intermediate and 7% low binding affinity), indicating that also TAP transport as predicted by TAPPred is similar between MiHA and reference peptides (sensitivity 40% and specificity 46% for high affinity threshold; sensitivity 92% and specificity 7% for intermediate affinity threshold by ROC analysis).

### Integration of HLA class I binding affinity, C-terminal proteasomal cleavage and TAP transport

Predictions for HLA class I binding affinity, C-terminal proteasomal cleavage and TAP transporter efficiency are integrated in the combined algorithm NetCTLpan 1.1. In the algorithm, the weight on HLA class I binding affinity, C-terminal proteasomal cleavage and TAP transport efficiency is 0.750, 0.225 and 0.025 by default, respectively. Using the default threshold for epitope identification (<1%-Rank), 44 (68%) of the 65 MiHA were predicted as epitopes, including 5 (33%) of the 15 HLA-A*02:01-restricted MiHA and 13 (72%) of the 18 HLA-B*07:02-restricted MiHA (Fig 2D). Notably, NetCTLpan 1.1 also failed to identify the 6 MiHA that were predicted as NB peptides by NetMHCpan 2.8. In the reference set of peptides, 21% of peptides with predicted binding to HLA-A*02:01 and 46% of peptides with predicted binding to HLA-B*07:02 were predicted as potential epitopes. These data demonstrate that HLA-B*07:02-restricted MiHA are more accurately predicted by NetCTLpan 1.1 (72% *versus* 46%, p = 0.0514) than HLA-A*02:01-restricted MiHA (33% *versus* 21%, p = 0.3338). ROC curves were plotted to determine the contribution of each algorithm to the overall predictive performance of the integrated algorithm of NetCTLpan 1.1 (S2A Fig) and to compare NetCTLpan 1.1 and NetMHCpan 2.8 (S2B Fig). The data demonstrated that MiHA cannot be more accurately characterized by an approach in which predicted C-terminal proteasomal cleavage and TAP transport are integrated with HLA class I binding affinity as compared to prediction tools for HLA class I binding affinity alone.

### *In vivo* immunogenicity

**Fig 3:**
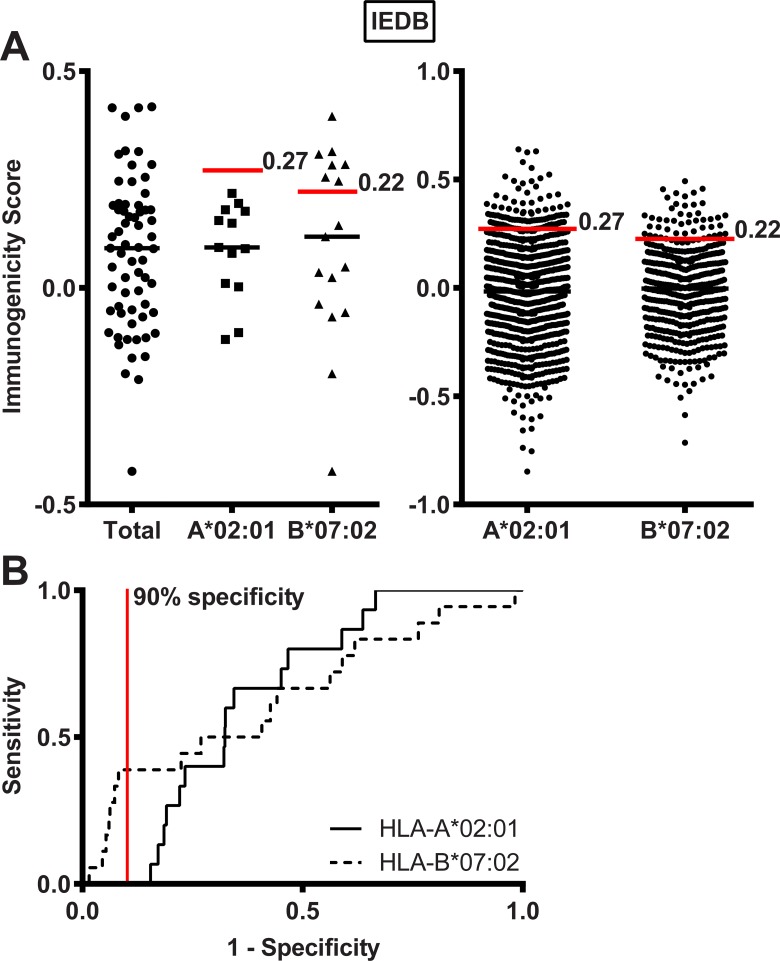
Predicted *in vivo* immunogenicity. (A) *In vivo* immunogenicity as predicted by the MHC I immunogenicity tool of the IEDB for the total group of MiHA (n = 65) and for MiHA with predicted binding to HLA-A*02:01 (n = 13) or HLA-B*07:02 (n = 17) by NetMHCpan 2.8. Indicated are immunogenicity scores for MiHA (left) and reference peptides (right). Designated are median immunogenicity scores (black horizontal lines) and thresholds of 0.27 and 0.22 to define immunogenic peptides for MiHA binding to HLA-A*02:01 or HLA-B*07:02, respectively (red lines). The data show a significant difference in proportion of immunogenic peptides between HLA-B*07:02-restricted MiHA and reference peptides (41% *versus* 10% with p = 0.0014 using Fisher’s exact test), but no significant difference between HLA-A*02:01-restricted MiHA and reference peptides (0% *versus* 10% with p = 0.3825 using Fisher’s exact test). (B) ROC curves for *in vivo* immunogenicity as predicted by the online tool of the IEDB for HLA-A*02:01 (solid line) and HLA-B*07:02 (dashed line) based on prediction data for MiHA and reference peptides. Thresholds with 90% specificity are indicated by the red vertical line.

The final step in the HLA class I pathway is antigen recognition by CD8 T-cells. The Immune Epitope Database and Analysis Resource (IEDB) has designed an online tool to predict *in vivo* immunogenicity of peptide antigens. Immunogenicity scores for the 65 MiHA in Table 2 were homogenously distributed with a range from -0.42 to 0.42 and a median score of 0.09. Individual values and median immunogenicity scores for the total set of MiHA as well as for MiHA with predicted binding to HLA-A*02:01 and HLA-B*07:02 and their reference peptides are shown in Fig 3A. Based on ROC curves as shown in Fig 3B, we determined thresholds with 90% specificity to predict *in vivo* immunogenicity of peptides binding to HLA-A*02:01 (>0.27) and HLA-B*07:02 (>0.22). Using the threshold of >0.27, none of the 13 HLA-A*02:01-restricted MiHA were predicted to be immunogenic as compared to 10% of the reference peptides. For HLA-B*07:02, however, 7 (41%) of the 17 antigens were predicted to be immunogenic using the threshold of >0.22 as compared to 10% of reference peptides. These data demonstrate that the MHC I immunogenicity tool of IEDB can be used to predict *in vivo* immunogenicity for peptides binding to HLA-B*07:02. However, it should be noted that classifying peptides into immunogenic and non-immunogenic peptides using a score >0.22 leads to a considerable number of HLA-B*07:02-restricted MiHA (59%) that are designated as non-immunogenic peptides, indicating that this threshold has a low sensitivity to characterize MiHA.

### *In silico* characteristics of MiHA and their allelic variants

MiHA arise as a result of SNP differences between patient and donor, which often lead to a change in a single non-synonymous amino acid between the MiHA as expressed on the patient cell and its allelic variant in the donor cell. Of the 65 HLA class I-restricted MiHA in Table 2, allelic variants do not exist for 8 MiHA (ACC-6, LB-ITGB2-1, LRH-1, PANE1, 3 MiHA encoded by *UGT2B17* and ZAPHIR). For the remaining 57 MiHA, allelic variants do exist. The majority of these allelic variants have not been identified as *in vivo* T-cell targets. This can be explained by insufficient searching for specific T-cells, but may also indicate that allelic variants cannot be processed or presented on the cell surface or that no specific TCRs are present in the naive repertoire of donor lymphocytes. Therefore, we explored whether MiHA differ from their allelic variants in *in silico* characteristics as determined by online prediction algorithms.

**Fig 4:**
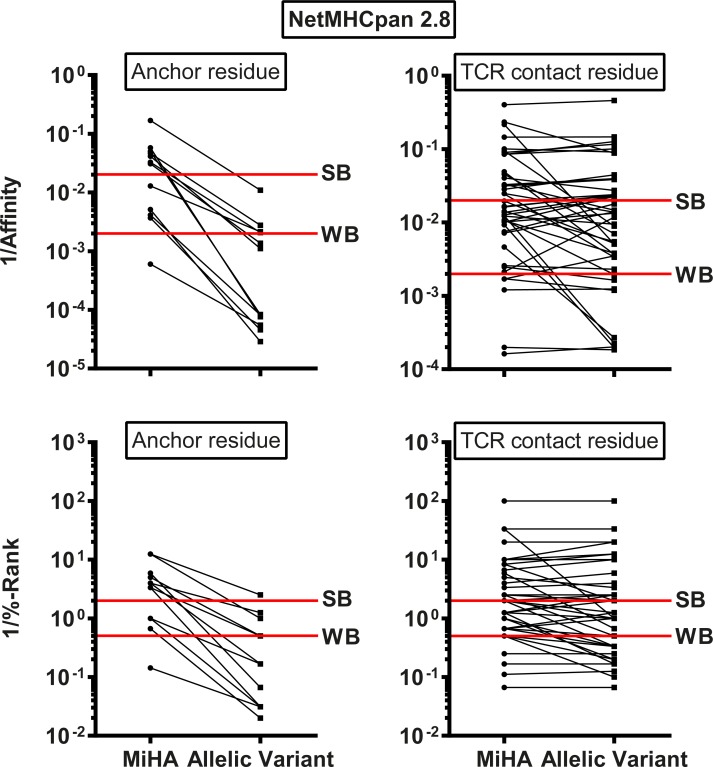
Predicted HLA class I binding affinity for MiHA and allelic variants. HLA class I binding affinity as predicted for MiHA and their allelic variants by NetMHCpan 2.8. Predicted affinity (1/affinity (nM); upper) and %-Rank (1/%-Rank; lower) are shown for all MiHA with allelic variants (n = 57) divided into two groups based on whether the polymorphic amino acid is an anchor residue (n = 12; left) or TCR contact residue (n = 45; right). Default thresholds for SB and WB peptides are indicated by red lines. The data show that predicted HLA class I binding for the 12 MiHA with polymorphic amino acids at anchor positions was significantly higher than for their allelic variants (p = 0.0005 using Wilcoxon signed rank test). For the MiHA with polymorphic amino acids at TCR contact residues (n = 45), predicted HLA class I binding as compared to their allelic variants was higher for 9 MiHA with the variant residue immediately adjacent to the anchor at position 2 (p = 0.0039 using Wilcoxon signed rank test), but similar for the remaining 36 antigens (p = 0.1965 using Wilcoxon signed rank test).

First, we examined and compared HLA class I binding affinity as predicted by NetMHCpan 2.8 between MiHA and their allelic variants (Fig 4). Of the 57 pairs of MiHA and allelic variants, two MiHA (ACC-1Y and HB-1H) have allelic variants that can be targeted by T-cells *in vivo*, indicating that these peptides are immunogenic in two directions. For these bi-allelic MiHA, the epitope that was first identified and published as *in vivo* T-cell target is indicated as MiHA and the counterpart is indicated as allelic variant. We divided the MiHA and their allelic variants into two groups based on whether the polymorphic amino acids are present at anchor positions or TCR contact positions. Anchor residue motifs that are used for HLA class I binding are shown in Table 3. HLA class I binding affinity was examined for 57 pairs of MiHA and allelic variants. Of these 57 pairs, 12 pairs contained polymorphic residues at anchor positions. As expected, for all pairs with polymorphic amino acids at anchor positions, predicted HLA class I binding affinity for MiHA was significantly higher than for their allelic variants (p = 0.0005). Of the 45 pairs with polymorphic amino acids at TCR contact residues, 9 MiHA had predicted HLA class I binding affinities that were significantly higher than their allelic variants (p = 0.0039). In all these peptides, the polymorphic amino acid was located immediately adjacent to the N-terminal anchor residue at position 2. For the remaining 36 pairs, predicted HLA class I binding affinity was similar between MiHA and allelic variants (p = 0.1965).

**Table 3:** Binding motifs for HLA class I alleles.

HLA-allele	Peptide length	Anchor residues
A*01:01	9–11	P_2_, P_3_, P_end_
A*02:01	9–11	P_2_, P_end_
A*03:01	9–11	P_2,_ P_3_, P_end_
A*24:02	9–11	P_2_, P_end_
A*29:02	9–11	P_end_
A*31:01	9–11	P_end_
A*33:03	9–11	P_end_
A*68:01	9–11	P_2_, P_end_
C*03:03	9–11	P_1_, P_2_, P_end_
B*07:02	9–11	P_2_, P_end_
B*08:01	9–11	P_3_, P_5_, P_end_
B*13:02	9–11	P_2_, P_3_, P_end_
B*15:01	9–11	P_2_, P_end_
B*40:01	9–11	P_2_, P_end_
B*40:02	9–11	P_2_, P_end_
B*44:02	9–11	P_2_, P_end_
B*44:03	9–11	P_2_, P_end_
B*51:01	9–11	P_2_, P_end_
B*57:01	9–11	P_2_, P_end_

Binding motifs as available at http://www.cbs.dtu.dk/biotools/MHCMotifViewer/Human_alleles.html [24].

We also compared MiHA and their allelic variants in predicted stability of the peptide-HLA class I complex by NetMHCstab 1.0 (S3A Fig). HLA class I restriction alleles were available for 41 pairs of MiHA and allelic variants. For 7 pairs with polymorphic amino acids at anchor residues, predicted stability of the peptide-HLA class I complex was significantly higher for MiHA than for their allelic variants (p = 0.0156), while predicted stability was similar for the majority of 34 pairs with polymorphic amino acids at TCR contact residues (p = 0.0781).

Finally, 57 pairs of MiHA and allelic variants were compared for predicted proteasomal cleavage, TAP affinity and *in vivo* immunogenicity (S3B–S3D Fig). No difference was observed in predicted proteasomal cleavage by NetChop 3.1 (81% for MiHA *versus* 81% for allelic variants, p = 1.000) and TAP affinity by TAPPred (40% high, 53% intermediate and 8% low affinity peptides for MiHA *versus* 47% high, 43% intermediate and 9% low affinity peptides for allelic variants). Moreover, immunogenicity scores as determined by the online tool of the IEDB were similar between MiHA (range between -0.42 and 0.42 with a median score of 0.09) and allelic variants (range between -0.54 and 0.46 with a median score of 0.05) (p = 0.2871). When a threshold of >0.25 was applied to define immunogenic peptides, 8 (14%) MiHA and 10 (18%) allelic variants were predicted to be immunogenic, including 5 pairs of MiHA and allelic variants for HLA-B*07:02.

In conclusion, the data show that predicted HLA class I binding affinity for 12 MiHA with polymorphic amino acids at anchor positions is significantly higher than for their allelic variants as well as for 9 MiHA with polymorphic amino acids at TCR contact residues in which the variant residue is located immediately adjacent to the anchor residue at position 2. The majority of MiHA (n = 36), however, do not differ from their allelic variants in *in silico* characteristics, indicating that these allelic variants can potentially be presented on the cell surface

## Discussion

MiHA can be identified by forward and reverse strategies. In forward strategies, T-cells from *in vivo* immune responses after alloSCT are used to identify the antigen, whereas peptides are used to search for specific T-cells in reverse strategies [4–6]. Particularly in reverse strategies, selection of candidate peptides by prediction algorithms for HLA class I binding affinity, stability of the peptide-HLA complex, proteasomal cleavage, TAP transport and *in vivo* immunogenicity may be relevant to increase the efficiency of MiHA discovery. To explore the value of online prediction algorithms, we determined the *in silico* characteristics of 68 autosomal HLA class I-restricted MiHA which have all been identified as natural T-cell ligands by forward strategies. As such, these MiHA should follow all rules for endogenous processing and presentation and antigen recognition by specific T-cells.

Of the 68 HLA class I-restricted MiHA that were analyzed, NetMHCpan 2.8 accurately predicted 38 (56%) antigens as SB peptides and 21 (31%) antigens as WB peptides. We also compared HLA class I binding affinity between MiHA and reference peptides and showed that the proportion of SB peptides is higher in the group of MiHA (54% for HLA-A*02:01 and 65% for HLA-B*07:02) than in the reference set (28% for HLA-A*02:01 and 24% for HLA-B*07:02). Using a more robust and quantitative approach, sensitivity and specificity were determined by ROC analysis for the default thresholds of NetMHCpan 2.8 based on prediction data for MiHA and reference peptides for HLA-A*02:01 and HLA-B*07:02. Our data showed that the threshold for SB has a high specificity but low sensitivity, whereas the threshold for WB has a high sensitivity but low specificity. This implies that in reverse strategies, selection of SB peptides has a high chance that the peptide is a true MiHA, but many MiHA will be missed, whereas selection of WB peptides has a low chance that true MiHA are missed, but the strategy is rather inefficient and many peptides need to be synthesized and screened.

Of the 9 antigens that were predicted as NB peptides, 3 antigens were excluded from further analyses, since experimental data confirming that the peptide sequences as reported in Table 1 are the actual minimal epitope are lacking. Of the remaining 6 NB antigens, we noticed that 5 (83%) antigens contained a cysteine residue as anchor (n = 2) or as residue adjacent to the anchor (n = 3). In contrast, 13 (23%) of the 59 antigens that were predicted as SB or WB peptides contained a cysteine residue and of these 13 antigens, only 6 antigens contained the cysteine as anchor (n = 1) or as residue adjacent to the anchor (n = 5). Since peptides with cysteine residues are highly underrepresented in databases used to train prediction algorithms for HLA class I binding, our data suggest that the accuracy of NetMHCpan 2.8 to predict HLA class I binding of cysteine containing peptides may be low.

NetMHCstab 1.0 is an algorithm that predicts stability of the peptide-HLA complex. We demonstrated that this algorithm failed to predict 10 (30%) of the 33 HLA-A*02:01- and HLA -B*07:02-restricted MiHA as stable peptide-HLA complexes. As such, MiHA are predicted as stable peptide-HLA complexes by NetMHCstab 1.0 with lower sensitivity than as HLA-binding peptides by NetMHCpan 2.8, which failed to predict 3 (9%) of these 33 MiHA. Specificity of NetMHCstab 1.0 as determined by ROC analysis, however, is high, illustrating that the chance that a peptide selected based on high predicted stability as determined by NetMHCstab 1.0 is a true MiHA is high. Unfortunately, only 13 HLA class I alleles are currently available in NetMHCstab 1.0 as compared to more than 2900 HLA class I alleles in NetMHCpan 2.8.

By comparing MiHA with reference peptides, we demonstrated that predicted proteasomal cleavage by NetChop 3.1 and predicted affinity for TAP by TAPPred was similar in both groups, suggesting that MiHA characterization cannot be improved by applying these algorithms. In NetCTLpan 1.1, predictions for HLA class I binding affinity, C-terminal proteasomal cleavage and TAP transporter efficiency are integrated in a combined algorithm with weights of 0.750, 0.225 and 0.025 by default, respectively. NetCTLpan 1.1 failed to identify the same 6 MiHA that were predicted as NB peptides by NetMHCpan 2.8, indicating that predicted C-terminal proteasomal cleavage and TAP transport affinity in the combined algorithm cannot compensate for weak HLA class I binding affinity. We also analyzed the predictive performance of NetCTLpan 1.1 by ROC analysis and demonstrated that the curve for the combined algorithm was similar as the curve for HLA class I binding affinity, illustrating that MiHA cannot be more accurately characterized by an approach in which predicted C-terminal proteasomal cleavage and TAP transport are integrated with HLA class I binding affinity as compared to prediction tools for HLA class I binding affinity alone.

The Immune Epitope Database and Analysis Resource (IEDB) has designed an online tool to predict *in vivo* immunogenicity of peptide antigens. We defined the thresholds for immunogenic peptides for MiHA binding to HLA-A*02:01 and HLA-B*07:02 by ROC analysis and demonstrated that the MHC I immunogenicity tool of IEDB can be used to predict *in vivo* immunogenicity of peptides binding to HLA-B*07:02. As such, selection of peptides with an immunogenicity score >0.22 may be considered as additional step to HLA class I binding prediction to improve discovery of HLA-B*07:02-restricted MiHA. The value of the online tool of IEDB has also been reported by Bassani-Sternberg et al. [29], who demonstrated that within the HLA-ligandome as analyzed by mass spectrometry, peptides that are known T-cell epitopes from cancer-associated proteins were more often predicted to be immunogenic than other HLA class I binding peptides from the same proteins. However, it should be emphasized that the sensitivity of the online tool of IEDB is low and that it failed to predict immunogenicity for 10 (59%) MiHA with predicted binding to HLA-B*07:02 as well as for all MiHA with predicted binding to HLA-A*02:01. It can be speculated that HLA-A*02:01-restricted epitopes allow more diversity in their TCR contact residues than HLA-B*07:02-restricted epitopes. This may explain why, despite use of many HLA-A*02:01-restricted epitopes for training of the algorithm, the sensitivity of the online tool of IEDB to predict *in vivo* immunogenicity of peptides binding to HLA-A*02:01 is lower than for HLA-B*07:02-binding peptides.

For the majority of HLA class I-restricted MiHA as shown in Table 2, allelic variants have not been identified as *in vivo* T-cell targets. This can be explained by insufficient searching for specific T-cells, but may also indicate that allelic variants cannot be processed or presented on the cell surface or that no specific TCRs are present in the naive repertoire of donor lymphocytes. To investigate processing and presentation of allelic variants, we determined whether the *in silico* characteristics as predicted by online algorithms are different between MiHA and their allelic variants. Our data showed that of the 57 pairs of MiHA and allelic variants that were analyzed, a minority of MiHA have amino acid substitutions at anchor positions (n = 12). As expected, HLA class I binding affinities for these MiHA are significantly higher than for their allelic variants. Predicted HLA class I binding affinity was also higher for a number of MiHA with amino acid substitutions at TCR contact residues (n = 9) in which the polymorphic residue is located immediately adjacent to the N-terminal anchor residue at position 2. For the majority of MiHA (n = 36), however, no difference in predicted HLA class I binding affinity was observed between MiHA and their allelic variants. Similar results were obtained for peptide-HLA class I complex stability as predicted by NetMHCstab 1.0 and also other prediction algorithms for proteasomal cleavage, TAP affinity and *in vivo* immunogenicity did not reveal any difference between MiHA and their allelic variants. Fritsch et al. [30] investigated 40 HLA class I-restricted neoantigens and also demonstrated that mutated amino acids are often present at TCR contact residues and that predicted HLA class I binding affinity is similar between mutated and native peptides. However, Duan et al. [31] showed in a reverse strategy for neoantigens that a relative score based on difference in HLA class I binding affinity as predicted by NetMHC 3.0 between mutant and wildtype peptides (differential agretopicity index; DAI) is superior in predicting *in vivo* immunogenicity in anti-tumor responses in mice than absolute values for HLA class I binding affinity as predicted for mutant peptides only. High DAI mostly resulted from amino acid substitutions at anchor residues between mutant and native peptides. Although it can be argued that peptides with amino acid changes at anchor positions may be more immunogenic as a result of lack of central tolerance, the majority of the 65 HLA class I-restricted MiHA that have been identified as *in vivo* T-cell targets in anti-tumor responses after alloSCT contain amino acid changes at TCR contact residues. Therefore, we recommend the use of prediction algorithms for HLA class I binding affinity for discovery of MiHA or neoantigens, but do not favor a strategy in which peptides are only selected for a difference in predicted HLA class I binding affinity between the two peptides as created by the genetic variants. Furthermore, since no evidence was obtained for improper processing or presentation for the majority of allelic variants, our data suggest that lack of *in vivo* immunogenicity of allelic variants is most likely due to insufficient searching for specific T-cells or absence of specific TCRs in the naive repertoire of donor lymphocytes.

In conclusion, our data showed that 87% of the HLA class I-restricted MiHA (56% SB and 31% WB peptides) were accurately predicted by NetMHCpan 2.8, but that besides HLA class I binding affinity, none of the other algorithms significantly contributed to MiHA characterization. Our results are relevant for discovery of T-cell ligands that are created by polymorphic (MiHA) or mutated (neoantigens) genetic variants.

## Supporting Information

S1 Dataset Tables showing the *in silico* analyses of MiHA, allelic variants and the reference set of peptides. (XLSX)

S1 Fig Comparison of predictive performance of NetMHCstab 1.0 and NetMHCcons 1.0. ROC curves for NetMHCstab 1.0 and NetMHCcons 1.0 for HLA-A*02:01 (left) and HLA-B*07:02 (right). Curves for NetMHCcons 1.0 (dashed line) and the integrated algorithm of NetMHCstab 1.0 (solid line) are plotted based on prediction data for MiHA and reference peptides. Sensitivity and specificity are indicated for default values for HS (>6 hrs) and WS (>2 hrs) complexes as predicted by NetMHCstab 1.0. For HLA-A*02:01, the AUC for NetMHCcons 1.0 and NetMHCstab 1.0 are 0.659 (p = 0.0341) and 0.596 (p = 0.2033), respectively. For HLA-B*07:02, the AUC for NetMHCcons 1.0 and NetMHCstab 1.0 are 0.763 (p = 0.0002) and 0.811 (p<0.0001), respectively. These data demonstrate that NetMHCstab 1.0 is slightly superior to NetMHCcons 1.0 for HLA-B*07:02, but not for HLA-A*02:01.
(TIF)

S2 Fig Predictive performance of NetCTLpan 1.1. (A) ROC curves for HLA class I binding affinity as predicted by NetMHCpan 2.3 (MHC; solid black line), TAP transport efficiency (TAP; solid grey line), C-terminal proteasomal cleavage as predicted by NetChop 3.0 (Cleavage; dashed line) and their combination (Combined; dotted line) are shown for HLA-A*02:01 (left) and HLA-B*07:02 (right). Graphs are plotted based on prediction data for MiHA and reference peptides. For HLA-A*02:01, the AUC for the MHC, TAP, Cleavage and Combined curves are 0.638 (p = 0.0663), 0.585 (p = 0.2598), 0.614 (p = 0.1295) and 0.646 (p = 0.0525), respectively. For HLA-B*07:02, the AUC for the MHC, TAP, Cleavage and Combined curves were 0.778 (p< 0.0001), 0.526 (p = 0.7082), 0.579 (p = 0.2539) and 0.760 (p = 0.0002), respectively. (B) ROC curves for NetCTLpan 1.1 (solid line) and NetMHCpan 2.8 (dashed line) are shown for HLA-A*02:01 (left) and HLA-B*07:02 (right). Graphs are plotted based on prediction data for MiHA and reference peptides. Sensitivity and specificity are indicated for the default value for epitope prediction (<1%-Rank) as used by NetCTLpan 1.1. For HLA-A*02:01, the AUC for NetCTLpan 1.1 and NetMHCpan 2.8 are 0.634 (p = 0.0758) and 0.625 (p = 0.0964), respectively. For HLA-B*07:02, the AUC for NetCTLpan 1.1 and NetMHCpan 2.8 are 0.737 (p = 0.0007) and 0.767 (p = 0.0001), respectively. The data show that MiHA cannot be more accurately characterized by NetCTLpan 1.1 in which C-terminal proteasomal cleavage and TAP transport efficiency are integrated with HLA class I binding affinity, as compared to prediction tools for HLA class I binding affinity alone.
(TIF)

S3 Fig Predicted stability of the peptide-HLA class I complex, proteasomal cleavage, TAP transport and *in vivo* immunogenicity for MiHA and allelic variants. (A) Peptide-HLA class I complex stability as predicted for MiHA and their allelic variants by NetMHCstab 1.0. Predicted half-life (hrs) is shown for all MiHA and allelic variants for which HLA class I restriction alleles are available in the algorithm (n = 41) divided into two groups based on whether the polymorphic amino acid is present at an anchor residue (n = 7; left) or TCR contact residue (n = 34; right). Default thresholds for HS and WS peptides are indicated by red lines. The data show that predicted peptide-HLA class I complex stability for the 7 MiHA with polymorphic amino acids at anchor positions was significantly higher than for their allelic variants (p = 0.0156 using Wilcoxon signed rank test), whereas predicted peptide-HLA class I complex stability was similar between MiHA and their allelic variants for the majority of 34 pairs with polymorphic amino acids at TCR contact residues (p = 0.0781 using Wilcoxon signed rank test). (B) Proteasomal cleavage at the C-terminus as predicted by NetChop 3.1 for all MiHA and their allelic variants (n = 53). Whole protein sequences were fed into the algorithm and default settings were used to predict proteasomal cleavage. Indicated are absolute numbers of peptides with predicted cleavage at the C-terminus. No significant difference was observed in proportion of peptides with predicted cleavage at the C-terminus between MiHA and allelic variants (81% for MiHA *versus* 81% for allelic variants, p = 1.000 using Fisher’s exact test). (C) Affinity for the TAP transporter as predicted by TAPPred with default settings for all MiHA and their allelic variants (n = 53). Indicated are absolute numbers of peptides with high (black bars), intermediate (light grey bars) and low (dark grey bars) affinity for TAP. No significant difference was observed in proportion of peptides with high or weak affinity for TAP between MiHA (40% high, 53% intermediate and 8% low affinity) and allelic variants (47% high, 43% intermediate and 9% low affinity). (D) *In vivo* immunogenicity as predicted by the MHC I immunogenicity tool of the IEDB for the total group of MiHA and allelic variants (n = 57). Median immunogenicity scores are indicated by black horizontal lines. Using a threshold of 0.25 (red line), no significant difference in proportion of immunogenic peptides was observed between MiHA (14%) and allelic variants (18%) (p = 0.798 using Fisher’s exact test).
(TIF)
