# Supplementary figures and images for: The Value of Online Algorithms to Predict T-Cell Ligands Created by Genetic Variants
Source: PLoS One. 2016 Sep 12;11(9):e0162808. doi: 10.1371/journal.pone.0162808 (PMC5019413; doi:10.1371/journal.pone.0162808)

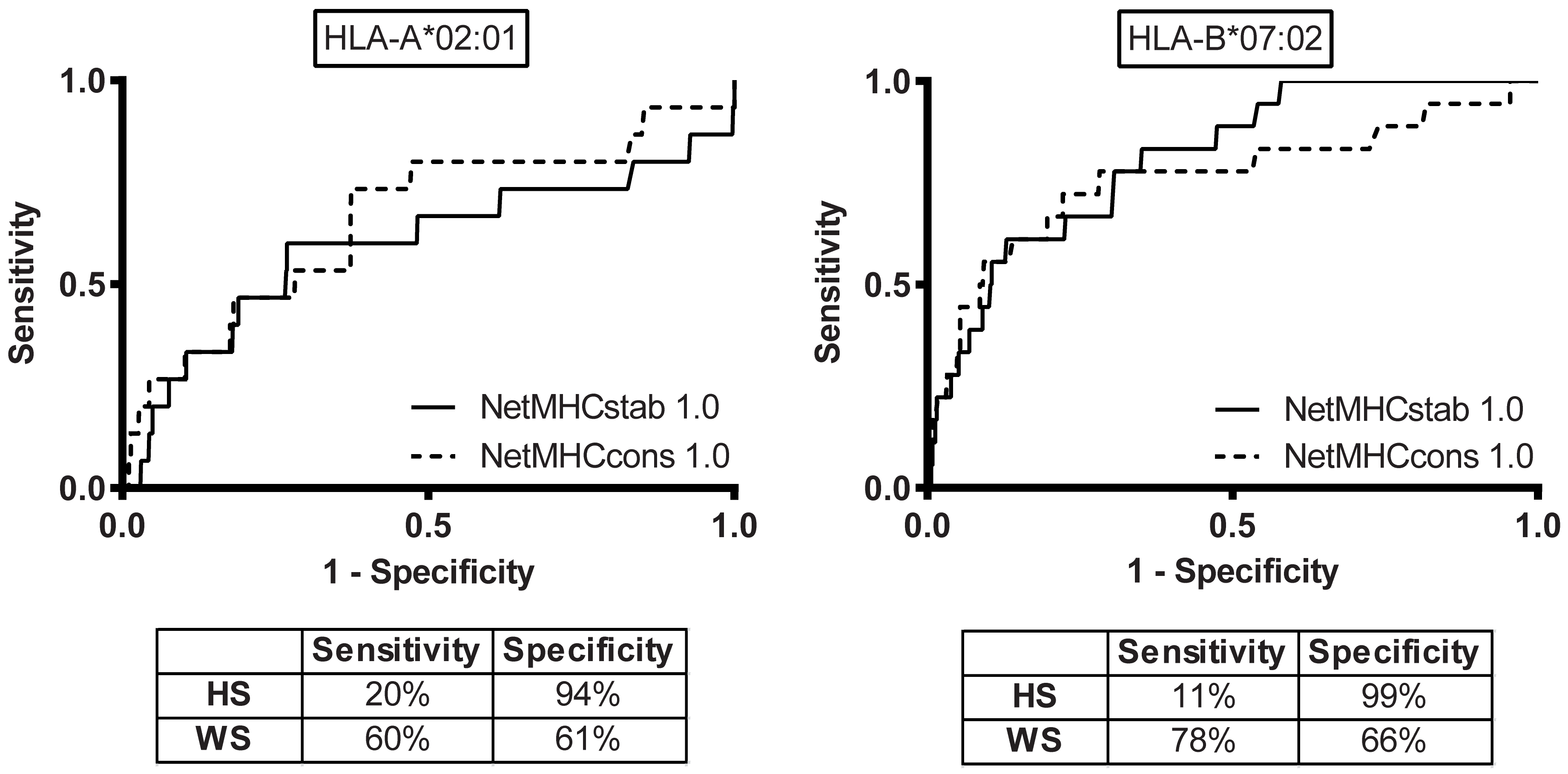

Supplement: S1 Fig — ROC curves for NetMHCstab 1.0 and NetMHCcons 1.0 for HLA-A*02:01 (left) and HLA-B*07:02 (right). Curves for NetMHCcons 1.0 (dashed line) and the integrated algorithm of NetMHCstab 1.0 (solid line) are plotted based on prediction data for MiHA and reference peptides. Sensitivity and specificity are indicated for default values for HS (>6 hrs) and WS (>2 hrs) complexes as predicted by NetMHCstab 1.0. For HLA-A*02:01, the AUC for NetMHCcons 1.0 and NetMHCstab 1.0 are 0.659 (p = 0.0341) and 0.596 (p = 0.2033), respectively. For HLA-B*07:02, the AUC for NetMHCcons 1.0 and NetMHCstab 1.0 are 0.763 (p = 0.0002) and 0.811 (p<0.0001), respectively. These data demonstrate that NetMHCstab 1.0 is slightly superior to NetMHCcons 1.0 for HLA-B*07:02, but not for HLA-A*02:01. (TIF) [file pone.0162808.s002.tif]

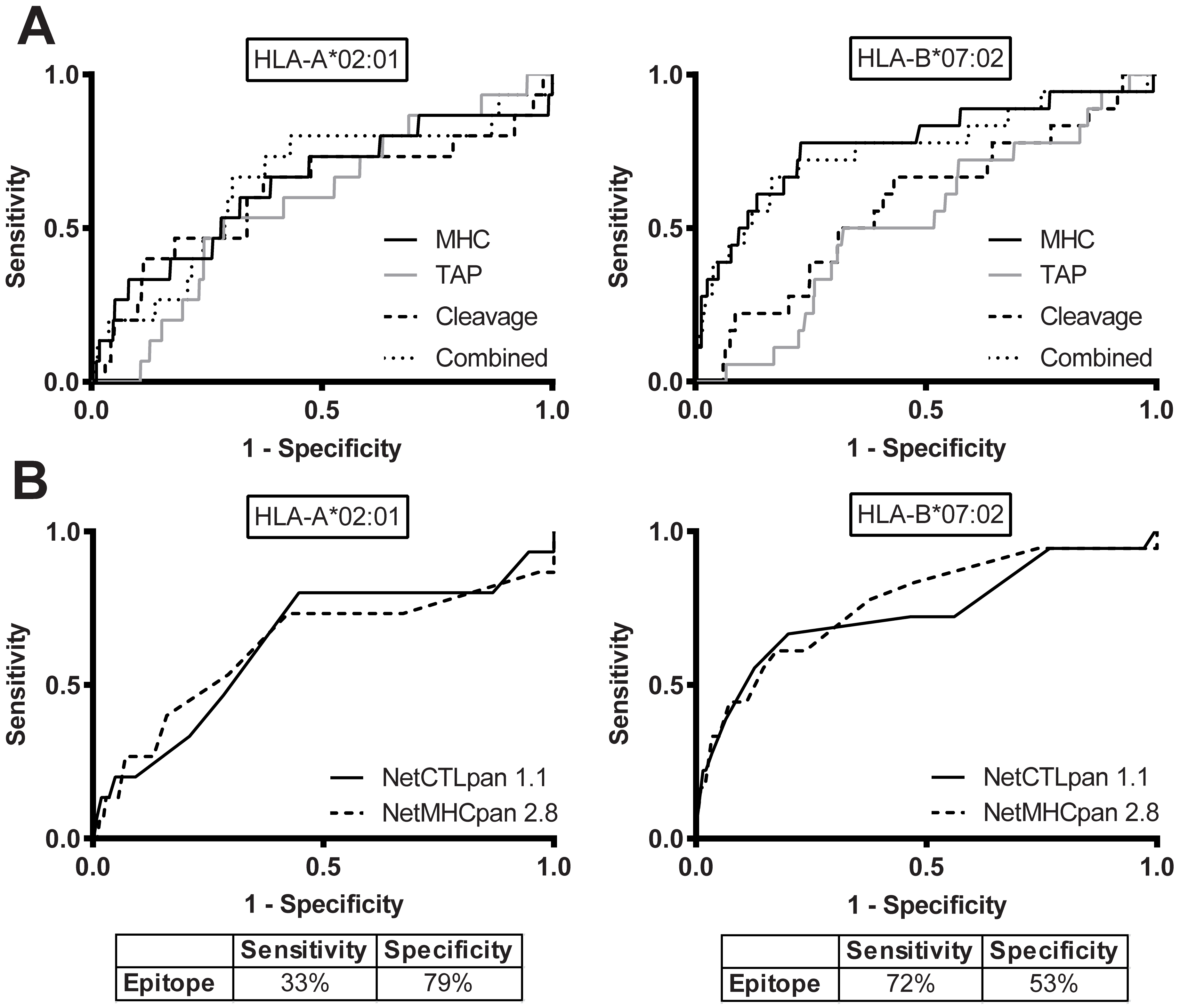

Supplement: S2 Fig — (A) ROC curves for HLA class I binding affinity as predicted by NetMHCpan 2.3 (MHC; solid black line), TAP transport efficiency (TAP; solid grey line), C-terminal proteasomal cleavage as predicted by NetChop 3.0 (Cleavage; dashed line) and their combination (Combined; dotted line) are shown for HLA-A*02:01 (left) and HLA-B*07:02 (right). Graphs are plotted based on prediction data for MiHA and reference peptides. For HLA-A*02:01, the AUC for the MHC, TAP, Cleavage and Combined curves are 0.638 (p = 0.0663), 0.585 (p = 0.2598), 0.614 (p = 0.1295) and 0.646 (p = 0.0525), respectively. For HLA-B*07:02, the AUC for the MHC, TAP, Cleavage and Combined curves were 0.778 (p< 0.0001), 0.526 (p = 0.7082), 0.579 (p = 0.2539) and 0.760 (p = 0.0002), respectively. (B) ROC curves for NetCTLpan 1.1 (solid line) and NetMHCpan 2.8 (dashed line) are shown for HLA-A*02:01 (left) and HLA-B*07:02 (right). Graphs are plotted based on prediction data for MiHA and reference peptides. Sensitivity and specificity are indicated for the default value for epitope prediction (<1%-Rank) as used by NetCTLpan 1.1. For HLA-A*02:01, the AUC for NetCTLpan 1.1 and NetMHCpan 2.8 are 0.634 (p = 0.0758) and 0.625 (p = 0.0964), respectively. For HLA-B*07:02, the AUC for NetCTLpan 1.1 and NetMHCpan 2.8 are 0.737 (p = 0.0007) and 0.767 (p = 0.0001), respectively. The data show that MiHA cannot be more accurately characterized by NetCTLpan 1.1 in which C-terminal proteasomal cleavage and TAP transport efficiency are integrated with HLA class I binding affinity, as compared to prediction tools for HLA class I binding affinity alone. (TIF) [file pone.0162808.s003.tif]

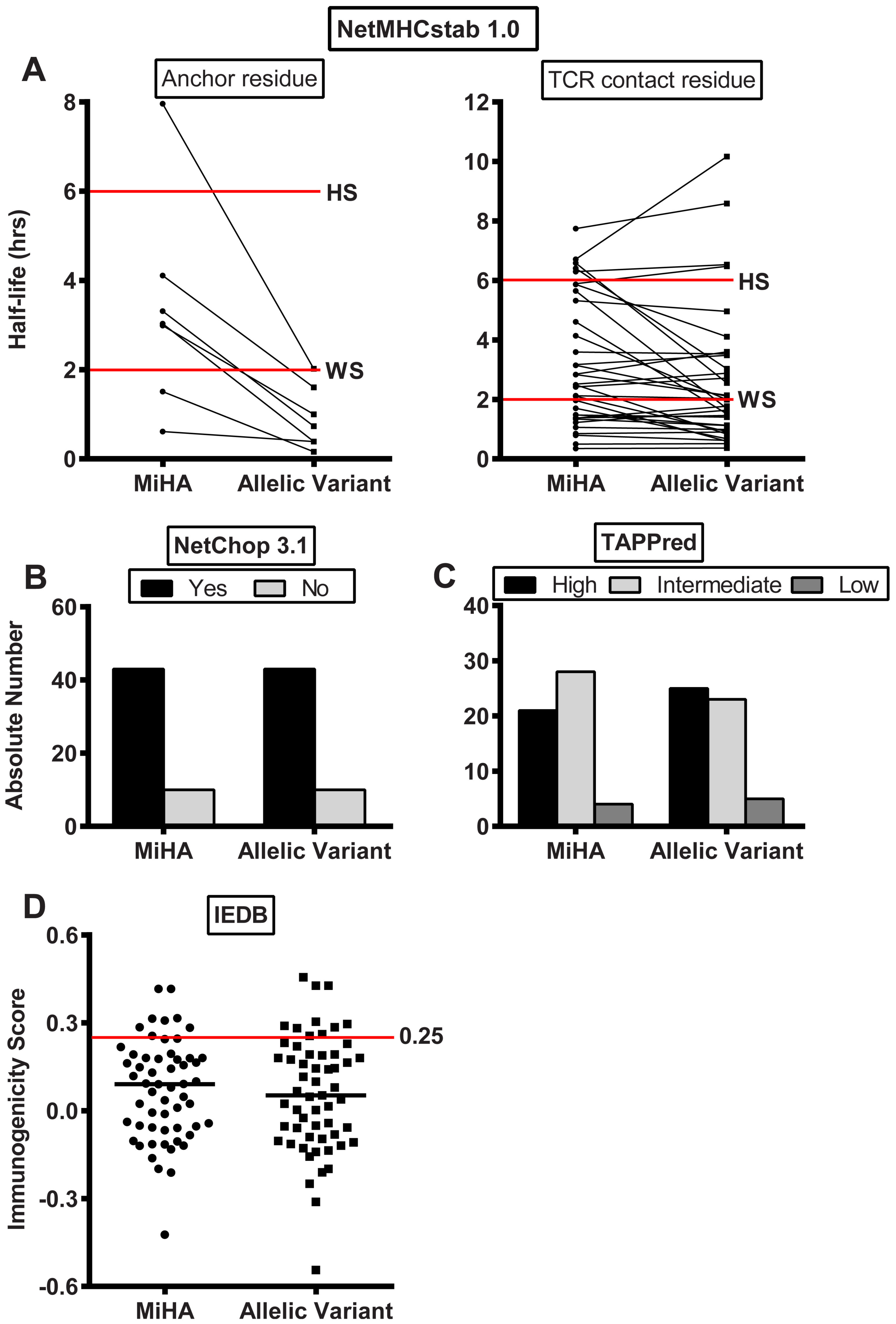

Supplement: S3 Fig — (A) Peptide-HLA class I complex stability as predicted for MiHA and their allelic variants by NetMHCstab 1.0. Predicted half-life (hrs) is shown for all MiHA and allelic variants for which HLA class I restriction alleles are available in the algorithm (n = 41) divided into two groups based on whether the polymorphic amino acid is present at an anchor residue (n = 7; left) or TCR contact residue (n = 34; right). Default thresholds for HS and WS peptides are indicated by red lines. The data show that predicted peptide-HLA class I complex stability for the 7 MiHA with polymorphic amino acids at anchor positions was significantly higher than for their allelic variants (p = 0.0156 using Wilcoxon signed rank test), whereas predicted peptide-HLA class I complex stability was similar between MiHA and their allelic variants for the majority of 34 pairs with polymorphic amino acids at TCR contact residues (p = 0.0781 using Wilcoxon signed rank test). (B) Proteasomal cleavage at the C-terminus as predicted by NetChop 3.1 for all MiHA and their allelic variants (n = 53). Whole protein sequences were fed into the algorithm and default settings were used to predict proteasomal cleavage. Indicated are absolute numbers of peptides with predicted cleavage at the C-terminus. No significant difference was observed in proportion of peptides with predicted cleavage at the C-terminus between MiHA and allelic variants (81% for MiHA versus 81% for allelic variants, p = 1.000 using Fisher’s exact test). (C) Affinity for the TAP transporter as predicted by TAPPred with default settings for all MiHA and their allelic variants (n = 53). Indicated are absolute numbers of peptides with high (black bars), intermediate (light grey bars) and low (dark grey bars) affinity for TAP. No significant difference was observed in proportion of peptides with high or weak affinity for TAP between MiHA (40% high, 53% intermediate and 8% low affinity) and allelic variants (47% high, 43% intermediate and 9% low [file pone.0162808.s004.tif]
